# High-Performance Temperature Sensors Based on Dual 4H-SiC JBS and SBD Devices

**DOI:** 10.3390/ma13020445

**Published:** 2020-01-17

**Authors:** Seong-Ji Min, Myeong Cheol Shin, Ngoc Thi Nguyen, Jong-Min Oh, Sang-Mo Koo

**Affiliations:** 1 Department of Electronic Materials Engineering, Kwangwoon University, 447-1 Wolgye-Dong, Nowon-Gu, Seoul 139-701, Korea; sjmin@kw.ac.kr (S.-J.M.); smc0753@naver.com (M.C.S.); ngochtdp@gmail.com (N.T.N.); jmOh@kw.ac.kr (J.-M.O.); 2 Department of Electrical and Mechanical Engineering, Nagoya Institute of Technology, Gokisocho, Showa Ward, Nagoya, Aichi 466-8555, Japan

**Keywords:** 4H-SiC diodes, junction barrier Schottky diode, Schottky barrier diode, high-temperature sensors, wide bandgap semiconductors

## Abstract

Schottky diode-based temperature sensors are the most common commercially available temperature sensors, and they are attracting increasing interest owing to their higher Schottky barrier height compared to their silicon counterparts. Therefore, this paper presents a comparison of the thermal sensitivity variation trend in temperature sensors, based on dual 4H-SiC junction barrier Schottky (JBS) diodes and Schottky barrier diodes (SBDs). The forward bias current–voltage characteristics were acquired by sweeping the DC bias voltage from 0 to 3 V. The dual JBS sensor exhibited a higher peak sensitivity (4.32 mV/K) than the sensitivity exhibited by the SBD sensor (2.85 mV/K), at temperatures ranging from 298 to 573 K. The JBS sensor exhibited a higher ideality factor and barrier height owing to the p–n junction in JBS devices. The developed sensor showed good repeatability, maintaining a stable output over several cycles of measurements on different days. It is worth noting that the ideality factor and barrier height influenced the forward biased voltage, leading to a higher sensitivity for the JBS device compared to the SBD device. This allows the JBS device to be suitably integrated with SiC power management and control circuitry to create a sensing module capable of working at high temperatures.

## 1. Introduction

Temperature sensors utilizing 4H-SiC-based diodes are advantageous in that they are highly sensitive and offer convenient interconnectivity with integrated circuits (ICs) [1,2]. To date, there have been several studies conducted regarding single-device 4H-SiC-based sensors for measuring elevated temperatures [3,4]. Silicon Schottky diodes are commonly used as temperature sensors owing to their cost-effectiveness and technological maturity. However, the physical properties of Si devices degrade quickly under high-temperature operations, making them less suitable for such uses. Due to its properties, including a higher Schottky barrier, SiC on the other hand is a very promising candidate for temperature sensing applications in high-temperature environments or in other harsh conditions [5,6,7]. However, SiC Schottky diodes suffer from reliability problems in the Schottky contact, in addition to a considerable leakage current as temperatures rise [8]. SiC p–n diodes can be a suitable alternative for operations at raised temperatures exceeding 600 °C, owing to their stability and the lower dependence of their saturation current on temperature [3]. Moreover, the low saturation current of the p–i–n diode, well below the forward biasing current at high temperatures, reduces the nonlinear effects in the *V_D_*–T characteristics, thus allowing the design and fabrication of highly linear sensors operating in a wider temperature range [9]. All of these reported simple devices show low sensitivities and relatively poor linearity. In a recent work, for the first time, a proportional to absolute temperature (PTAT) sensor was developed, based on two identical 4H-SiC Schottky diodes biased with different currents [10]. However, there have hitherto been no reports on sensitivity variations in SiC junction barrier Schottky (JBS) devices. To the best of our knowledge, this is the first study comparing the behaviors of dual 4H-SiC JBS and Schottky barrier diode (SBD) devices used for thermal sensing in the temperature range between 298 and 573 K. First, detailed analyses of the forward characteristics versus temperature (*I*–*V*–*T*) characteristics, considering several optional variations in barrier height and ideality factor with temperature, indicated defects at the interface between the metal and the semiconductor in the manufactured devices. Second, the thermal sensitivity (*S*) of the fabricated devices was extracted from the slope of the plot of the differential forward voltage across the two diodes, which were biased at different constant currents, versus temperature. Finally, sensor characteristics were studied and the reasons for the higher sensitivity of the JBS diode (compared to the SBD) were determined.

## 2. Device Structure and Characteristics

### 2.1. Device Structure

**Figure 1:**
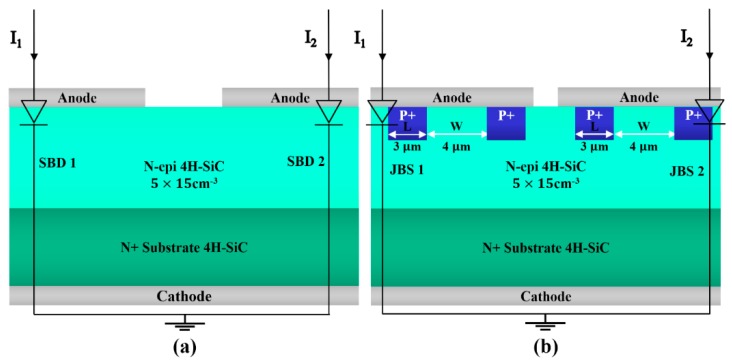
(**a**) PTAT sensor circuit and schematic cross sections of integrated 4H-SiC Schottky barrier diodes (SBDs) and (**b**) 4H-SiC junction barrier Schottky (JBS) diodes.

Figure 1 shows a schematic cross section of the investigated 4H-SiC-based SBD and JBS diodes. Both types were fabricated on 12 μm thick and n-doped (*N* = 5 × 10^15^ cm^−3^) epitaxial layers grown on 325 μm thick, n^+^-type 4H-SiC substrates, each with an estimated active area of 2.56 mm^2^. The sensor chips of the SBD and JBS devices each contain two diodes, which show nearly identical forward bias current–voltage (*I*−*V*) characteristics, as they were manufactured on the same substrate and epi-layer.

### 2.2. I–V Characteristics

The forward voltage of the device as a function of current is governed by the thermionic emission (TE) theory, which can be expressed using the standard TE model as follows Equation (1):

(1) $$V_{SBD}=R_{SBD}I+\frac{kT\eta_{SBD}}{q}\ln\left(\frac{I}{AR^{**}T^{2}}\right)$$

(2) $$V_{JBS}=R_{JBS}I+\frac{kT\eta_{JBS}}{q}\ln\left(\frac{P+S}{S-2W}\frac{I}{AR^{**}T^{2}}\right)$$

**Figure 2:**
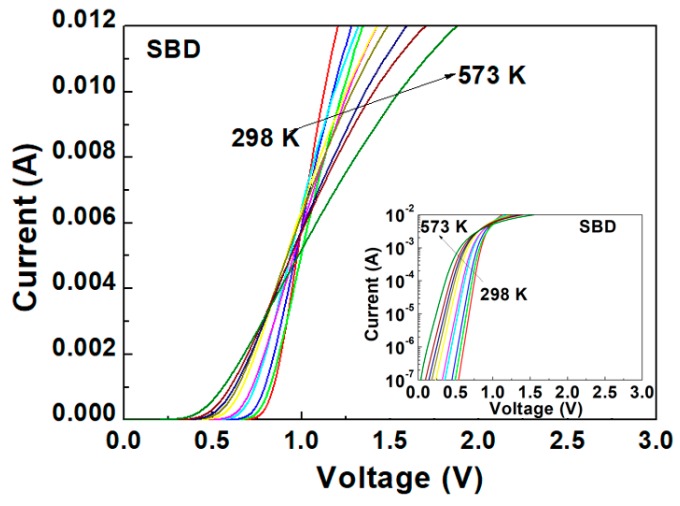
Forward *I*–*V* characteristics of SBD in the temperature range from 298 to 573 K.

**Figure 3:**
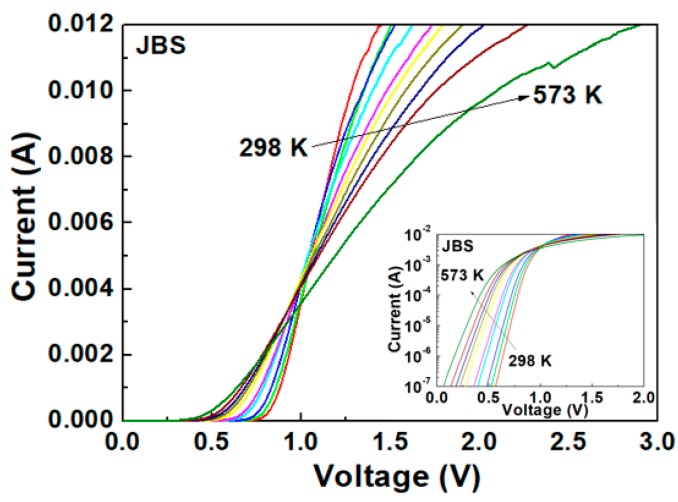
Forward *I*–*V* characteristics of JBS device in the temperature range from 298 to 573 K.

The *I*–*V* characteristics were measured at discrete temperatures, after stabilization, ranging from 298 to 573 K. These characteristics were obtained by sweeping the DC bias voltage from 0 to 3 V with steps of 1 mV. A Keithley 4200 source meter was used to measure the *I*–*V* curves of the devices. Figure 2 and Figure 3 present the forward characteristics versus temperature (*I*–*V*–*T*) plots for the SBD and JBS diode in the range of 0–0.012 A, respectively.

As expected, the curves cross at the same *I*–*V* point, known as the zero temperature coefficient (ZTC) point, typical of Schottky diodes [11]. This ZTC point divides the groups of curves into two regions: a first region featuring a positive temperature coefficient of *I_D_*, and second region featuring a negative temperature coefficient of *I_D_*.

For gate voltages above the ZTC point, the drain current density decreases with an increasing temperature due to the negative temperature coefficient. Devices are considered thermally stable when operating under this regime [12]. For gate voltages below the ZTC point, a local increase in heat generation is caused by the positive temperature coefficient of the current density. A temperature hot spot can then be created if the transient thermal resistance is too high to immediately disperse the heat. This leads to thermal instability, resulting in localized thermal runaway, and ultimately device destruction [13]. The drain current temperature coefficient αT = Δ*I_D_*/ΔT is used as an electrical indicator of transient thermal resistance and as a measure of the ZTC [12,13]. As shown in Figure 2 and Figure 3, the JBS diode displays a lower ZTC point (4 mA) compared to that of the SBD (6 mA). Effectively, it appears that the JBS diode reduces the transient thermal resistance in high-temperature operations, thereby exhibiting better thermal stability than the SBD.

The larger turn-on voltage of the JBS diode is because of the built-in voltage of the p–n junction. The temperature increase causes a reduction in the turn-on voltage for both the SBD and JBS diode. The shift in the turn-on voltage with respect to temperature is well elucidated by the TE theory [14].

### 2.3. Variations in Barrier Height and Ideality Factor with Temperature

As observed in Equations (1) and (2), variations in the temperature and current influence the forward voltage and, consequently, the device’s thermal sensitivity. To explain the measured thermal sensitivity of fabricated devices, a systematic study on the variations in the barrier height and ideality factor with temperature is required.

From *I*–*V*–*T* measurements, it is possible to extract the corresponding ideality factor, *η*, and barrier height, *Φ*. In particular, *Φ* and *η* were calculated from the intercept with the vertical axis and from the slope of the graph showing the *ln*(*I_D_*)–*V_D_* characteristics, respectively.

**Figure 4:**
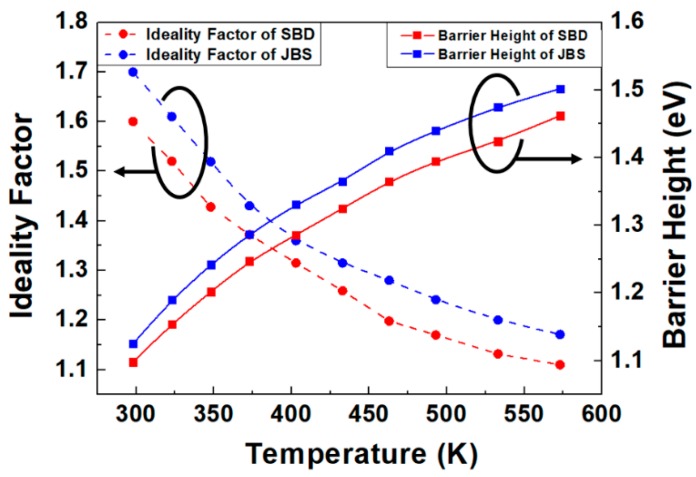
Variations in the ideality factor and barrier height of the SBD and JBS diode as functions of temperature.

The experimental *I*–*V*–*T* data presented in Figure 4 show *Φ* and *η* as functions of temperature. As can be seen in this figure, η decreases while *Φ* increases with an increase in temperature. It has been proposed that this could be because of the tunneling of charge carriers through the barrier, the generation of a recombination current in the depletion region, an uneven barrier height distribution at the metal–semiconductor interface, or oxidation [15,16].

The ideality factor for the JBS diode decreases from 1.66 to 1.17, while that for the SBD decreases from 1.4 to 1.1. The barrier height of the JBS diode increases from 1.12 to 1.49, while that of the SBD increases from 1.15 to 1.52.

The barrier height of the JBS diode is higher than that of the SBD, owing to the additional p–n junction in the JBS diode structure. The height of the Schottky barrier is smaller than that of the barrier in the p–n junction. In a p–n junction, the height of the barrier that separates electrons in the conduction band of the n-type region from the bottom of the conduction band in the p-type region is roughly equivalent to the energy gap. The barrier height in Schottky devices is approximately two-thirds of the energy gap or less [17].

The higher ideality factor of the JBS diode can be attributed to different sources. The P+ region of the SiC JBS diode is formed by ion implantation and annealing, which cause interface states, residual defects, and surface roughening in JBS diodes, resulting in a larger ideality factor [18]. The ideality factor increases with increasing doping concentrations [19]. The JBS diode with a P+ region has higher doping levels than the SBD, thus resulting in a higher ideality factor.

JBS diodes comprise p–n and Schottky diodes. Moreover, for an ideal Schottky diode, *η* is approximately 1 owing to no recombination in the depletion layer; in contrast, the *η* value of a p–n junction ranges from 1.2 to 2 owing to recombination in the depletion layer [20]. This results in *η* being higher for a JBS diode than for an SBD.

## 3. Diode Temperature Sensor

### 3.1. Thermal Sensitivity

The JBS diode and SBD have identical *I*−*V* characteristics for each type of diode with our setup. They were biased via two known currents, *I_D_*_1_ and *I_D_*_2_ (Figure 1), that were maintained as constants over the complete working temperature range. The difference between the diode voltages (*V_D_*_2_ − *V_D_*_1_) is related to temperature *T* as per the following equation:

(3) $$V_{D2}-V_{D1}=R_{s}(I_{D2}-I_{D1})+\frac{kT\eta }{q}\ln\left(\frac{I_{D2}}{I_{D1}}\right)$$

The sensor sensitivity, *S*, can be obtained from the slope of the plot showing the difference between the forward voltages across the two diodes biased at different constant currents, versus temperature, i.e., the (*V*_D2_ − *V*_D1_)–*T* plot.

**Figure 5:**
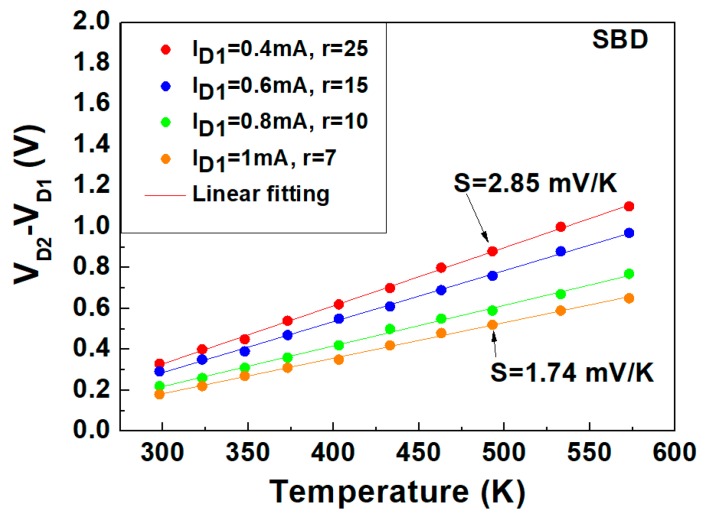
Measured (symbols) and modeled (lines) voltage difference vs. temperature profiles at different bias currents (*I_D_*_1_) and current ratios (*r* = *I_D_*_2_/*I_D_*_1_).

**Figure 6:**
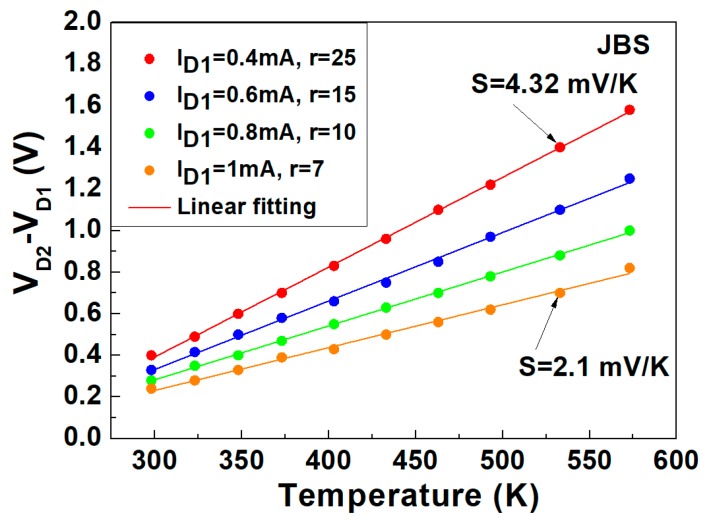
Measured (symbols) and modeled (lines) voltage difference vs. temperature profiles at different bias currents (*I_D_*_1_) and current ratios (*r* = *I_D_*_2_/*I_D_*_1_).

The profiles of (*V_D_*_2_ − *V_D_*_1_) versus *T* at specific current ratios (*r* = *I_D_*_2_*/I_D_*_1_ of 7, 10, 15, and 25), simultaneously measured in the temperature range of 298 to 573 K, are shown in addition to the linear fitting model for the SBD (Figure 5) and JBS diode (Figure 6). The sensitivity defined by the slope of the (*V_D_*_2_ − *V_D_*_1_) vs. *T* plot is higher at the higher ratios of the current levels.

The coefficient of determination (*R^2^*) [21] was calculated to quantify the linearity goodness by fitting the experimental data using Equation (4) as follows:

(4) $$R^{2}=1-\frac{\sum_{i=1}^{n}{\left(V_{D,i}-f_{L,i}\right)}^{2}}{\sum_{i=1}^{n}{\left(V_{D,i}-{\bar{V}}_{D}\right)}^{2}}$$

where *n* is the number of the experimental points, *V_D_*_,*i*_ and *f_L_*_,*i*_ are obtained through actual measurements and the best linear fit model, respectively, at temperature *i*, and ${\bar{V}}_{D}$ is the average of all the measurements.

The voltage difference is an increasing function of temperature as observed in Equation (3). All the characteristics of the SBD and JBS diode show a good degree of linearity (*R*^2^ > 0.999) for *I_D_*_1_, ranging from 0.4 to 1 mA with different current ratios, *r*.

As observed in Figure 5, when *I_D_*_1_ is 1 mA and *r* is 7, the sensitivity of the SBD is 1.74 mV/K, and it increases for higher *r* and lower *I_D_*_1_ values. When *I_D_*_1_ is 0.4 mA and *r* = 25, the sensitivity is 2.85 mV/K. Here, the SBD shows maximum linearity with *R*^2^ = 0.9996, corresponding to an rms error of ±0.8 K.

According to Figure 6, when *I_D_*_1_ is 1 mA and *r* is 7, the sensitivity of the JBS diode is 2.1 mV/K and monotonically increases to 4.32 mV/K for *I_D_*_1_ = 0.4 mA and *r* = 25. The maximum linearity is shown at *R*^2^ = 0.99962 for *S* = 4.32 mV/K with an rms error of ±0.78 K.

In the considered wide bias current range from 10 μA to 10 mA, the sensitivity of the JBS diode is always higher than that of the SBD. The higher sensitivity of the JBS diode can be attributed to the higher ideality factor of the JBS diode compared to the SBD. Moreover, the sensitivity of p–n diodes is higher than that of Schottky diodes, and the JBS diode amalgamates the advantages of p–n and Schottky diodes [3,5].

### 3.2. Sensor Linearity

**Figure 7:**
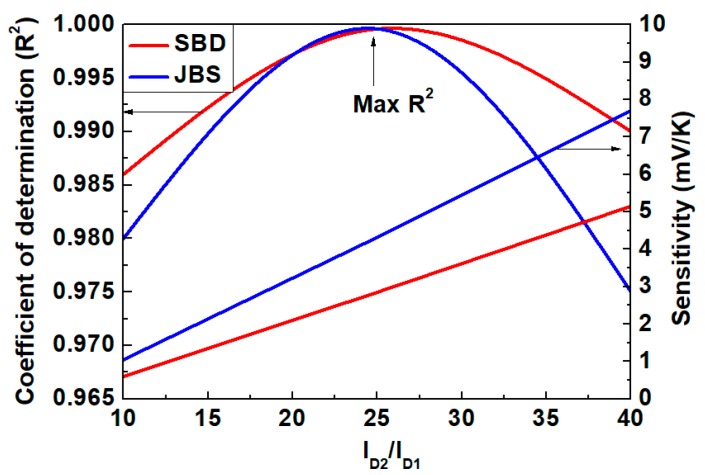
Coefficient of determination and sensitivity vs. current ratio for *I_D_*_1_ = 0.4 mA, calculated in the temperature range of 298 to 573 K.

Figure 7 presents a comparison of both *R*^2^ and *S* for the SBD and JBS diodes, with different values of *r* at a forward current (*I_D_*_1_) of 0.4 mA. Over the considered temperature range, the *R*^2^ value of the SBD varies by 0.52% from an average value of 0.992, while that of the JBS diode varies by 0.88% from an average value of 0.985. The reduction in the *R*^2^ value for the JBS diode can be attributed to the effect of series resistance due to ohmic contact (in the P^+^ region at the metal–semiconductor junction).

After reaching their maximum values at *r* = 25, the *R*^2^ values of both the SBD and JBS diode decrease. Above the crossing point shown in Figure 2 and Figure 3 (SBD and JBS, respectively) with high current levels, the series resistance (*R_s_*) drop term of Equation (3) is not negligible. As observed in Figure 7, a low *R*^2^ value shows that *R_s_* is nonlinear with respect to *T* at high currents.

Below the crossing point (ZTC), where R_s_ (*I_D_*_2_ − *I_D_*_1_) is negligible, the outputs of the SBD and JBS sensors show a decline in *R*^2^ owing to the nonlinear behavior of the ideality factor, *η*(*T*), with respect to *T*. High sensitivity to temperature with the maximum linearity of the SBD and JBS sensors can be achieved by exploiting the tradeoff between the nonlinear behaviors of *R_s_* and *η* with respect to temperature. The best tradeoff is obtained at *r* = 25, with the maximum *R*^2^ being 0.9996 and *S* = 2.85 mV/K for the SBD, whereas the maximum *R*^2^ is 0.9995 with *S* = 4.32 mV/K for the JBS diode.

**Figure 8:**
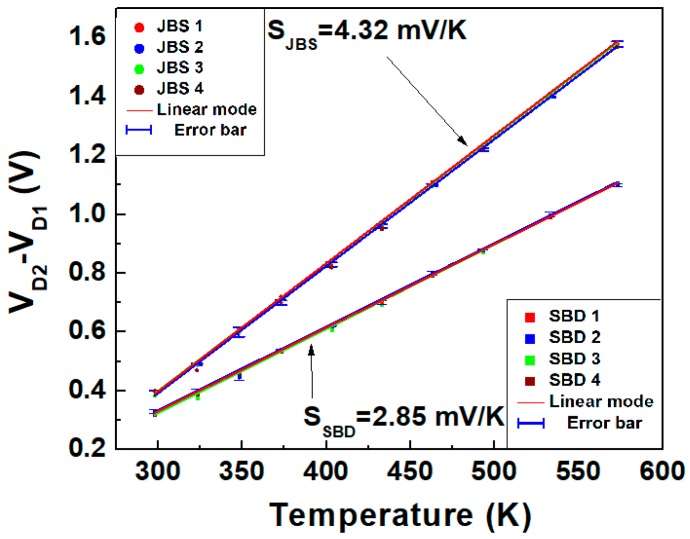
Linear fit and rms errors of the (*V_D_*_2_ − *V_D_*_1_)–*T* characteristics for four dual JBS diodes and SBDs fabricated using the same process and measured on different days. The bias currents are the same for all sensors, *I_D_*_1_ = 0.4 mA and *r* = 25.

Furthermore, to evaluate the long-term stability and performance reproducibility, four different dual JBS diodes and dual SBDs were tested by repeating the same cycles of measurements iteratively, from 298 to 573 K, on different days. Figure 8 displays the results obtained, which show that the calculated maximum errors of the JBS diode and SBD are lower than ±2% and ±2.5%, respectively. JBS diodes show a sensitivity of 4.32 mV/K, which is higher by 51.6% compared to that of the SBD.

## 4. Conclusions

In summary, a comprehensive study was carried out to compare the sensitivity of dual JBS and SBD temperature sensors. The dual JBS diodes showed the higher peak sensitivity, of 4.32 mV/K, compared to the 2.85 mV/K of the SBD at a forward current ratio (*I_D_*_2_/*I_D_*_1_) of 25. In addition, the JBS diode exhibited a higher ideality factor and barrier height, owing to the p–n junction in the JBS diode. Moreover, the same cycles of measurements performed on different days were iterated for four dual 4H-SiC JBS diodes and SBDs, showing long-term stability and good output repeatability. Therefore, dual 4H-SiC JBS diodes are more suitable for designing and fabricating highly linear sensors operating in a wide temperature range.
